# Enhancing Membrane Permeability of Fluorescein-Type Chromophore Through Covalent Attachment of Chlorinated Dodecaborate

**DOI:** 10.3390/molecules29225416

**Published:** 2024-11-17

**Authors:** Hibiki Nakamura, Satoshi Yamamoto, Yumiko K. Kawamura, Taro Kitazawa, Mutsumi Kimura, Yu Kitazawa

**Affiliations:** 1 Department of Chemistry and Materials, Faculty of Textile Science and Technology, Shinshu University, Ueda 386-8567, Japanmkimura@shinshu-u.ac.jp (M.K.); 2 Department of Molecular Biology and Genetics, Aarhus University, Universitetsbyen 81, 8000 Aarhus, Denmark; 3 Research Initiative for Supra-Materials (RISM), Shinshu University, Ueda 386-8567, Japan

**Keywords:** boron cluster, chaotropic anion, membrane transport

## Abstract

Anionic boron clusters, such as [B_12_X_12_]^2−^ (X = Cl, Br, I), have attracted attention in pharmaceuticals due to their unique superchaotropic properties. In particular, [B_12_Br_12_]^2−^ (**1**) has demonstrated strong interactions with biomolecules, facilitating cargo translocation across plasma membranes. In this study, we investigated the effect of covalently attaching chlorinated dodecaborate moiety [B_12_Cl_11_O-]^2−^ to 6-carboxyfluorescein (6-FAM) (**3**) via a PEG3 linker to form conjugate (**4**). We compared the membrane permeability of this covalent conjugate with that of non-covalent interactions between 6-FAM (**3**) and [B_12_Cl_12_]^2−^ (**2**). Live-cell fluorescence imaging revealed that the covalent conjugate exhibited enhanced membrane permeability and water solubility while maintaining low cytotoxicity. These results highlight the potential of covalent conjugation with boron clusters for improving the cellular uptake of hydrophilic cargos.

## 1. Introduction

**Figure 1:**
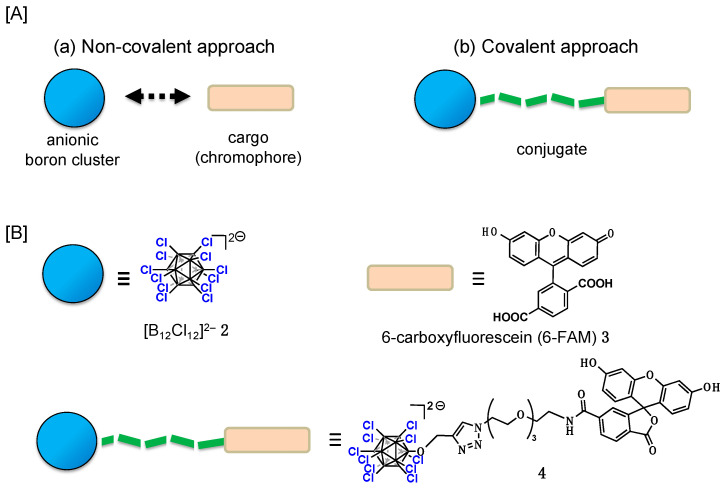
(**A**) This work: comparison of cell membrane permeability between (**a**) non-covalent approach and (**b**) covalent approach. (**B**) Molecular design in this work.

Anionic boron clusters are recognized for their broad applications, especially in pharmaceuticals, due to their distinctive chemical properties. Notably, closo-borates such as [B_12_X_12_]^2−^ and [B_10_X_10_]^2−^ (X = H, Cl, Br, I), as well as metallacarborane clusters, including cobalt bisdicarbollides (COSANs), have gained significant attention owing to recent findings regarding their unique physicochemical properties, particularly their superchaotropic nature [1,2,3,4,5,6,7,8,9,10,11,12,13]. Superchaotropic anions are known for their strong interactions with biomolecules and lipid membranes, leading to the development of novel membrane transport mechanisms based on this ionic character [6,7,8,9,10,11,12,13]. One of the representative examples is [B_12_Br_12_]^2−^ (**1**), which has been recognized as an efficient carrier for a variety of biomolecules, including both cationic and neutral species [6]. Since this novel concept obviates the traditional amphiphilic transport mechanism and overcomes the limitations of amphiphilic transporters, the development of chaotropic membrane transport carriers is of great importance. Mechanistically, the boron cluster first interacts with the cargo through enthalpy-driven chaotropic interactions, leading to the desolvation of the hydrophilic cargo and the lowering of the energy barrier required for membrane translocation. Once the cargo passes through the membrane non-disruptively, it dissociates from the boron cluster due to the reversible nature of these chaotropic interactions, facilitating cytosolic cargo release. Given the importance of the interaction between the cargo and the boron cluster for translocation of the cargo, we hypothesized that covalently linking the boron cluster to the cargo could enhance the permeability of the cargo (Figure 1A).

Regarding the conjugation of chromophores with superchaotropic boron clusters, COSAN conjugates with BODIPY chromophores have been reported to exhibit cellular uptake, and similar results have been reported for COSAN conjugates with organotin compounds [14,15,16]. These studies suggest that the covalent attachment of boron clusters to cargos can enhance cellular uptake. It is noteworthy that Nau and coworkers reported a fluorescein-substituted perbrominated dodecaborate cluster as an anchor dye for large macrocyclic hosts and explored its application in indicator displacement assays [17]. However, direct comparisons between covalent and non-covalent conjugation approaches remain limited in the previous reports. Since [B_12_Cl_12_]^2−^ (**2**) has lower chaotropicity than [B_12_Br_12_]^2−^ (**1**) but still functions as a chaotropic membrane carrier [6,10], we chose to use it in our study to directly compare covalent and non-covalent approaches, while also exploring the potential of **2**. Herein, we explore the impact of covalently attaching [B_12_Cl_11_O-]^2−^ to 6-carboxyfluorescein (6-FAM) (**3**) through a PEG3 linker to form conjugate (**4**), assessing its membrane translocation properties in comparison with the non-covalent approach using 6-FAM and [B_12_Cl_12_]^2−^ (**2**) (Figure 1B). Our results demonstrate that covalent attachment enhances the membrane permeability and solubility of 6-FAM while maintaining low cytotoxicity, highlighting the potential of this strategy for drug delivery applications.

## 2. Results

### 2.1. Synthesis

**Figure 2:**
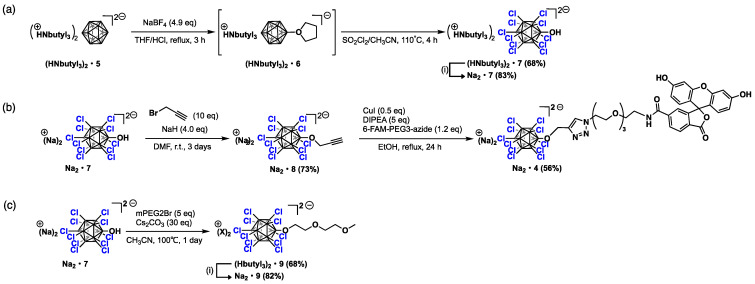
(**a**) Improved synthesis of **7**; (**b**) synthetic route to conjugate **4**; (**c**) synthesis of **9** (i) NaOH (10 eq), H_2_O, 50 °C, 12 h.

We commenced our study by developing an improved synthesis of [B_12_Cl_11_OH]^2−^ (**7**). In seminal work by Hawthorne, [B_12_H_11_OH]^2−^ was synthesized by the hydroxylation of [B_12_H_12_]^2−^ (**5**) in sulfuric acid [18]. During the synthesis, **5** undergoes a sequential and regioselective acid-catalyzed hydroxylation. Although this is a high-yielding and efficient method, precise control of time, acid concentration, and temperature is crucial to prevent the formation of over-hydroxylated products, such as [B_12_H_10_(OH)_2_]^2−^. We reported a high-yield, gram-scale synthesis of [B_12_Br_11_OH]^2−^ via the bromination of a dodecaborate containing a cyclic oxonium group, [B_12_H_11_O(CH_2_)_4_]^2−^ (**6**), in a one-pot reaction starting from the parent [B_12_H_12_]^2−^ (**5**) [19]. In this reaction, the acid-mediated cleavage of the C–O bond of **6** during the bromination step generates highly pure [B_12_Br_11_OH]^2−^ without the formation of any over-hydroxylated products such as [B_12_Br_10_(OH)_2_]^2−^. We wondered if this protocol could be applicable as an alternative method for the synthesis of [B_12_Cl_11_OH]^2−^ (**7**) (Figure 2a). Thus, **6** was prepared according to the literature method, confirmed using ^1^H NMR spectroscopy, and directly chlorinated in SO_2_Cl_2_/MeCN, based on the report by Duttwyler [20]. Chlorination was conducted using pressure tubes (Ace) at 110 °C. It is noteworthy that while the reaction vessels were plugged tightly with Teflon screw caps, the O-rings originally provided were replaced by wrapping PTFE tape around the lower part of the screw cap’s thread. This modification helped prevent overpressure from the reaction. Although some of the volatile chlorine gas and sulfur dioxide generated during the chlorination reaction escaped from the reaction vessels, chlorination of the boron cluster proceeded smoothly. After the reaction, [B_12_Cl_11_OSO_2_Cl]^2−^ and [B_12_Cl_11_OSO_2_OH]^2−^, likely formed via the reaction between the hydroxyl group of the boron cluster and SO_2_Cl_2_, were detected by ESI-TOF-MS. These were successfully converted to the desired product, [B_12_Cl_11_OH]^2−^, by boiling the mixtures in water. It should be noted that tributylammonium salts [HNbutyl_3_]_2_**•7** were used instead of tetrabutylammonium salts to facilitate the subsequent counter-cation exchange to sodium cation via the deprotonative decomposition of the tributylammonium cation by sodium hydroxide. Overall, [HNbutyl_3_]_2_**•7** was obtained in 68% yield, starting from [HNbutyl_3_]_2_**•5** in a one-pot reaction, and subsequent counter cation exchange yielded pure Na_2_**•7**.

Starting from **7**, the boron cluster conjugate of fluorescein **4** was prepared in three steps based on the reported procedure (Figure 2b) [17]. The alkoxylation of [B_12_Cl_11_OH]^2−^ was performed in a basic medium (NaH and DMF) using propargyl bromide. The linkage of the fluorescein-type chromophore to the boron cluster was achieved by azide–alkyne click reaction. The introduction of mPEG2 was achieved by treating **7** with the corresponding alkyl bromide under basic conditions in acetonitrile using Cs_2_CO_3_ as a base (Figure 2c). The purification of *O*-mPEG2 **9** was unexpectedly problematic due to its high solubility in water; the hydrophilic impurities probably formed from mPEG2Br could not be removed by chromatography. After extensive trials, we found that the tributylammonium salts of **9** are insoluble in water, allowing for successful purification by washing with water, followed by filtration to yield pure [HNbutyl_3_]_2_**•9**. Notably, the triethylammonium salts of **9** are soluble in water, preventing purification by washing with water. Following a counter-cation exchange from the tributylammonium cation to sodium salts, Na_2_**•9** was obtained with an 82% yield starting from [HNbutyl_3_]_2_**•9**.

### 2.2. Cell Viability Assay and Membrane Translocation in Living Cells

**Figure 3:**
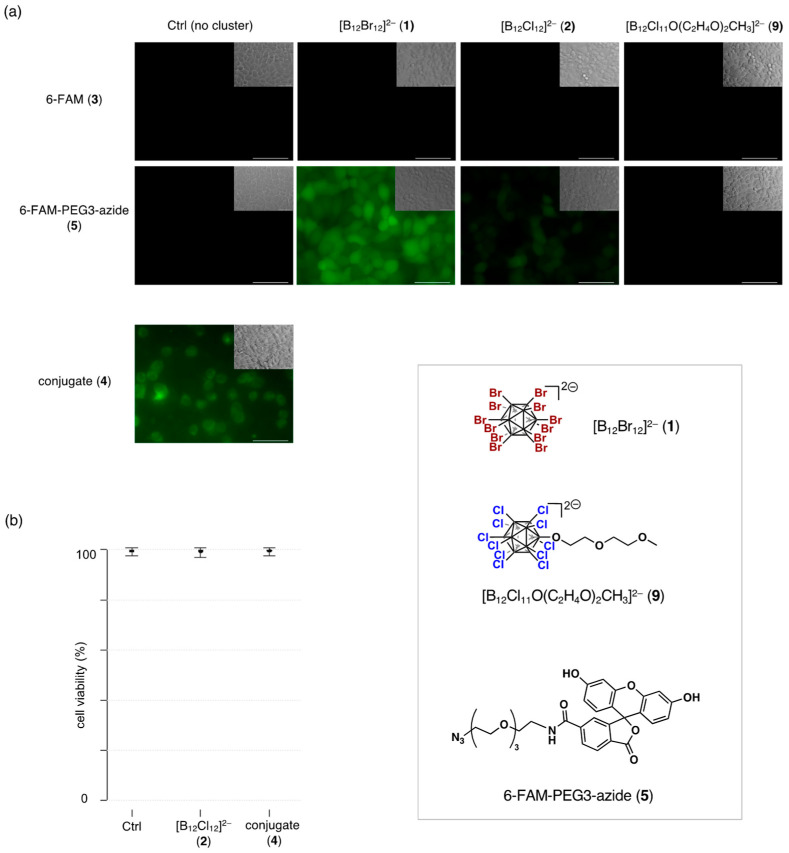
(**a**) (**Top**) HeLa cells were incubated with 20 μM 6-FAM **3** or 6-FAM-PEG-Azide **5** in the absence (Ctrl) or presence of 50 μM boron clusters (**1**, **2** or **9**) for 1.5 h. (**Bottom**) HeLa cells were incubated with 20 μM conjugate **4** for 1.5 h. Representative images of three biological replicates. The bright-field view is displayed at the top right of each panel. Scale bars, 50 μm; (**b**) HeLa cells were incubated with 20 μM boron clusters with the indicated concentration for 1.5 h, and cell viability was assessed after 24 h. Live cell number is divided by total cell number. Data are mean ± standard deviation of three biological replicates. No significant difference in viability among groups was found with one-way analysis of variance (ANOVA).

Cellular uptake of fluorescein derivatives was assessed in HeLa cells through live-cell fluorescence imaging (Figure 3a). The cellular uptake of 6-FAM **3**, either in the absence of the clusters or in the presence of [B_12_Br_12_]^2−^ (**1**) or [B_12_Cl_12_]^2−^ (**2**), was almost undetectable. This observation is consistent with previous reports, which suggest that translocation of negatively charged cargo using the superchaotropic boron cluster [B_12_Br_12_]^2−^ (**1**) is hindered due to the charge repulsion between the anionic cargo and the anionic boron cluster, limiting their interaction and preventing effective desolvation of the cargo [6]. Since 6-FAM **3** exists as an anionic species at physiological pH, it remains negatively charged, which likely hinders its translocation.

We also investigated the effects of introducing a PEG3 moiety to the fluorescein chromophore on its transmembrane behavior. Although 6-FAM-PEG3-azide **5** did not exhibit significant cellular uptake in the absence of boron clusters, diffuse fluorescence was detected in both the cytosol and the nucleus of the cells in the presence of [B_12_Br_12_]^2−^ (**1**). [B_12_Cl_12_]^2−^ (**2**) also facilitated detectable cellular retention. These results indicate that once the cargo is translocated inside the cell, intracellular retention occurs, consistent with previous reports [6,8,9,10,12,13]. Hence, it is suggested that low cellular uptake rather than retention is the primary reason why **3** does not accumulate in cells. The enhanced membrane permeability of 6-FAM-PEG3-azide in **5** compared to **3** can be attributed to the introduction of the PEG3 moiety. Firstly, the incorporation of the PEG3 spacer results in the amidation of one of the two carboxyl groups of 6-FAM, converting it from a dianionic species to a monoanionic species at physiological pH. This decrease in negative charge attenuates the electrostatic repulsion between the anionic cargo and the anionic boron cluster, thereby facilitating their interaction. Secondly, superchaotropic anions are known to exhibit an affinity for synthetic and biological interaction sites [11]. It is possible that the PEG3 moiety serves as a flexible hydrophilic spacer, enhancing the interaction between the fluorescein chromophore and the boron clusters, improving desolvation, and promoting direct translocation across the cell membrane.

In this context, we hypothesized that covalently linking the fluorescein chromophore to boron clusters would enhance the translocation of the cargo. Indeed, the internalization of conjugate **4** was observed more efficiently than the permeation of 6-FAM-PEG3-azide **5** facilitated by [B_12_Cl_12_]^2−^ (**2**), and much more efficiently than 6-FAM **3**, which could not cross the cell membrane even with the assistance of [B_12_Br_12_]^2−^ (**1**). The successful translocation of [B_12_Cl_11_O-]^2−^ conjugate **4** demonstrates the potential efficiency of the conjugation strategy. Previous studies suggest that monosubstituted brominated dodecaborates such as [B_12_Br_11_O-]^2−^ maintain their chaotropic properties [17]. For instance, they show an affinity for γ-cyclodextrin (γ-CD), albeit with a lower binding affinity than [B_12_Br_12_]^2−^, likely due to steric hindrance introduced by the linker. It is possible that the [B_12_Cl_11_O-]^2−^ moiety in **4** retains enough chaotropic properties to promote direct translocation. Further investigation into the translocation mechanism of **4** is crucial for future studies. For comparison, we also evaluated the transmembrane transport properties of boron clusters with a PEG moiety. However, neither 6-FAM **3** nor 6-FAM-PEG3-azide **5** showed translocation in the presence of **9**, likely due to two factors: (1) the PEG2 moiety of **9** does not sufficiently promote interaction between the boron cluster and the fluorescein chromophore, and (2) the hydrophilic PEG2 moiety diminishes the chaotropic properties of the chlorinated dodecaborate.

Regarding cytotoxicity, conjugate **4** did not exhibit significant cytotoxic effects at concentrations of up to 20 μM after 24 h (Figure 3b). The combination of membrane permeability and minimal cytotoxicity observed for conjugate **4** at the tested concentrations suggests its potential for further investigation as a drug delivery system (DDS) carrier.

### 2.3. Impact of Covalent Conjugation on Solubility

During the membrane translocation assay, we observed that conjugate **4** exhibited enhanced water solubility compared to the parent 6-FAM **3**. Water solubility is a crucial factor in drug design. While a representative strategy to improve solubility involves introducing hydrophilic moieties, these often result in reduced cell membrane permeability. Therefore, developing a method that enhances both cell permeability and water solubility is highly significant. Boron clusters with superchaotropic anionic properties are generally known for their high water solubility [5], and we anticipated that attaching a boron cluster would improve the solubility of the cargo molecule. Notably, 6-FAM **3** showed poor water solubility (<1 mM), whereas 6-FAM-azide **5**, possessing a PEG3 moiety, showed improved solubility (8 mM), likely due to the hydrophilicity of the PEG3 unit. As expected, the boron-cluster-conjugated **4** showed enhanced water solubility (>80 mM).

## 3. Materials and Methods

### 3.1. Instrumentation

NMR spectra were recorded on a BRUKER AVANCE NEC400 OneBay. Chemical shifts are expressed in *δ* (ppm) values. ^1^H NMR spectra were referenced to the peaks of the residual amounts of the (partially) protonated solvent as the internal standard. ^11^B NMR spectra were unreferenced. ^13^C NMR spectra were referenced to the solvent signal as the internal standard. The following abbreviations are used: s = singlet, d = doublet, t = triplet, m = multiplet, and bs = broad singlet. IR spectra were recorded on a Shimadzu Corporation IR Prestige-21 spectrometer. ESI mass spectra were measured on a Bruker micrOTOF-II-SF spectrometer. Melting points were determined on a Yanaco micro melting point machine. Normal-phase column chromatography was performed with Biotage Isolera One 1SW.

### 3.2. Materials

Unless otherwise noted, materials were purchased from Aldrich Inc. (St. Louis, MO, USA), Wako Pure Chemical Industries, Ltd. (Osaka, Japan), Tokyo Kasei Co. (Tokyo, Japan), and other commercial suppliers, and were used after appropriate purification. [Na]_2_[B_12_Br_12_^2−^] ((Na)_2_**•1**) was prepared according to the literature procedure [21]. Air- and moisture-sensitive manipulations were performed with standard Schlenk techniques. Normal-phase column chromatography was performed with silica gel 60 (230–400 mesh) from Merck.

### 3.3. Experimental Procedures

#### 3.3.1. Procedure for One-Pot Synthesis of [Na^+^]_2_[B_12_Cl_11_OH^2-^] (Na)_2_**•7**

(HNbutyl_3_)_2_**•5** (500 mg, 0.97 mmol), NaBF_4_ (522 mg, 4.76 mmol), THF (66 mL), and HCl (prepared by mixing 1 vol of 37% HCl in water with 3 vol of THF) (1.9 mL) were placed in an Ace flask. The reaction vessels were plugged tightly with Teflon screw caps, and the O-rings originally provided were replaced by wrapping PTFE tape around the lower part of the screw cap’s thread. The solution was stirred at 90 °C for 3 h. The generation of the intermediate (**2**) was checked by ^1^H NMR [19]. The solvent was removed under vacuum. SO_2_Cl_2_ (9.0 mL) and 3 mL of acetonitrile were added, and the solution was stirred at 110 °C for 4 h. The solvent was removed under vacuum, and the resulting material was again dissolved in water and stirred at 100 °C for 6 h. The precipitate was filtered off, washed with water, and dried in air to give (HNbutyl_3_)_2_**•7** (powder A). Tributylammonium chloride (643.4 mg, 2.91 mmol) was added to the filtrate, and the precipitate was filtered off, washed with water, and dried in air to give (HNbutyl_3_)_2_**•3** (powder B). The powder A and B were combined (600 mg, 68% yield based on (HNbutyl_3_)_2_**•5**).

The exchange of the counter cation to sodium cation (Na⁺) was performed as follows: (HNbutyl_3_)_2_**•7** (500 mg, 0.55 mmol), NaOH (219 mg, 5.49 mmol), and H_2_O (200 mL) were placed in a flask and stirred at 50 °C for 12 h. The mixture was then filtered to remove unreacted (HNbutyl_3_)_2_**•7**. The filtrate was neutralized using 10% aqueous HCl and evaporated to dryness to yield a white solid. This solid was washed with dichloromethane to remove tributylamine. The precipitate was collected by filtration and dried under vacuum to obtain a solid. This solid was dissolved in acetone and filtered to remove any insoluble materials, such as NaCl, and the filtrate was washed with dichloromethane to further remove tributylamine. Additionally, column chromatography on silica gel (eluent: CH_2_Cl_2_/MeOH) was performed to ensure the complete removal of NaCl and other impurities. The final solution was evaporated to dryness and dried under vacuum to give pure (Na)_2_**•7** (266 mg, 83% yield based on (HNbutyl_3_)_2_**•7**).

##### *[HNbutyl_3_^+^]_2_[B_12_Cl_11_OH^2–^] (HNbutyl_3_)_2_***•7** 

Brown solid; Yield: 72% mp; 179 °C; ^11^B{H}-NMR (128 MHz, acetone-*d_6_*) δ –7.66 (s, 1B), –13.50 (s, 11B): ^1^H{^11^B}-NMR (400 MHz, Acetone-*d_6_*) δ 3.39 (s, 12H), 1.87 (s, 12H), 1.45 (q, J = 7.5 Hz, 12H), 0.98 (t, J = 7.4 Hz, 18H) ^13^C-NMR (101 MHz, acetone-*d_6_*) δ 52.82, 28.99, 25.35, 19.68, 13.08 HR-ESI-TOF-MS *m*/*z* 268.3659 (calcd *m*/*z* 268.3615 for B_12_Cl_11_OH [M]^2−^).

##### *[Na^+^]_2_[B_12_Cl_11_OH^2−^] (Na)_2_***•7** 

White solid; Yield: 83% 221 °C; ^11^B{H}-NMR (128 MHz, acetone-*d_6_*) δ –7.73 (s, 1B), –13.50 (s, 11B):HR-ESI-TOF-MS *m*/*z* 268.3631 (calcd *m*/*z* 268.3615 for B_12_Cl_11_OH [M]^2−^).

#### 3.3.2. Procedure for the Synthesis of [Na^+^]_2_[B_12_Cl_11_OC_3_H_3_^2−^] (Na)_2_**•8**

(Na)_2_**•7** (165.8 mg, 0.33 mmol), NaH (32.0 mg, 1.32 mmol), propargyl bromide (392 mg, 3.3 mmol), and DMF (12 mL) were placed in a Schlenk tube and stirred at room temperature for 3 days. The solvent was removed under vacuum, and the solid was washed with water and hexane, and dried in air to give pure (Na)_2_**•8** (150 mg, 73% yield).

##### *[Na^+^]_2_[B_12_Cl_11_OC_3_H_3_^2^^−^] (Na)_2_***•8** 

White solid; Yield: 73% 209 °C; ^11^B{H}-NMR (128 MHz, acetone-*d_6_*) δ –8.06 (s, 1B), –14.09 (bs, 11B): ^1^H{^11^B}-NMR (400 MHz, acetone-*d_6_*) δ 4.81 (s, 2H), 2.58 (s, 1H): HR-ESI-TOF-MS m/z 287.3898 (calcd *m*/*z* 287.3855 for B_12_Cl_11_C_3_H_3_O [M]^2−^).

#### 3.3.3. Procedure for the Synthesis of (Na)_2_**•4**

A mixture of (Na)_2_**•7** (17 mg, 0.028 mmol), 6-FAM-PEG3-azide (20 mg, 0.034 mmol), diisopropylethylamine (18.1 mg, 0.14 mmol), and CuI (2.7 mg, 0.014 mmol) in EtOH (10 mL) was heated under reflux for 24 h using an oil bath. The reaction mixture was subsequently cooled to room temperature and the solvent was removed under reduced pressure. The crude product was purified by column chromatography on silica gel using CH_2_Cl_2_/CH_3_OH (5:1) as eluent to give pure (Na)_2_**•4** (12.2 mg, 56% yield).

##### *(Na)_2_***•4** 

Orange solid; Yield: 56% mp: 182 °C; ^11^B{H}-NMR (128 MHz, D_2_O) δ –7.79 (s, 1B), –14.05 (s, 11B); ^1^H{^11^B} NMR (400.00 MHz, acetone-*d_6_*) δ 8.04 (d, J = 8.0 Hz, 1H), 7.98 (d, J = 8.0 Hz, 1H), 7.87 (s, 1H), 7.70 (s, 1H), 7.22 (d, J = 9.3 Hz, 2H), 6.79 (d, J = 4.5 Hz, 3H), 6.76 (s, 1H), 4.90 (s, 2H), 4.42 (s, 2H), 3.78 (t, J = 4.6 Hz, 1H), 3.74 (t, J = 5.1 Hz, 1H), 3.60–3.65 (m, 4H), 3.54 (t, J = 3.1 Hz, 4H), 3.48 (s, 2H); ^13^C-NMR (101 MHz, D_2_O) δ 173.44, 169.44, 157.65, 146.82, 141.71, 135.12, 132.19, 131.60, 129.10, 127.98, 124.99, 120.89, 114.98, 103.34, 69.85, 69.72, 69.58, 68.83, 59.54, 50.05, 39.99 HR-ESI-TOF-MS *m*/*z* 575.1948 (calcd *m*/*z* 575.1951 for C_26_H_26_B_12_Cl_11_N_4_O_8_ [M]^2−^).

#### 3.3.4. Procedure for the Synthesis of [Na^+^]_2_[B_12_Cl_11_O(C_2_H_4_O)_2_CH_3_^2−^] (Na)_2_**•9**

(Na)_2_**•7** (240.0 mg, 0.412 mmol), Cs_2_CO_3_ (4105.3 mg, 12.6 mmol), methyl-PEG2-bromide (467.6 mg, 2.06 mmol), and acetonitrile (10 mL) were placed in a Schlenk tube and stirred at 100 °C for 1 day. The solvent was removed under vacuum, and the solid was dissolved in water, and tributylammonium chloride (911 mg, 4.12 mmol) was added. The resulting white precipitate was filtered off, washed with water, and dried in air to give pure (HNButyl_3_)_2_**•9** (283.4 mg, 68% yield).

The exchange of the counter cation to sodium cation (Na⁺) was performed as follows: (HNbutyl_3_)_2_**•9** (200 mg, 0.19 mmol), NaOH (79 mg, 1.97 mmol), and H_2_O (10 mL) were placed in a flask and stirred at 50 °C for 12 h. The mixture was then filtered to remove unreacted (HNbutyl_3_)_2_**•9**. The filtrate was neutralized using 10% aqueous HCl and evaporated to dryness to yield a white solid. This solid was washed with dichloromethane to remove tributylamine. The precipitate was collected by filtration and dried in air to obtain a solid. This solid was dissolved in acetone and filtered to remove any insoluble materials, and the filtrate was washed with dichloromethane to further remove tributylamine. The final solution was evaporated to dryness and dried under vacuum to give pure (Na)_2_**•9** (107 mg, 82% yield based on (HNbutyl_3_)_2_**•9**).

##### *[HNbutyl_3_^+^]_2_[B_12_Cl_11_O(C_2_H_4_O)_2_CH_3_^2−^]* *(HNbutyl_3_)_2_***•9** 

White solid; Yield: 68% mp: 188 °C; ^11^B {^1^H } NMR (128.00 MHz, acetone-*d_6_*) δ –7.5 (1B), –13.6 (11B) ^1^H{^11^B} NMR (400.00 MHz, acetone-*d_6_*) δ 7.79 (s, 1H), 4.15 (t, J = 6.0 Hz, 2H), 3.62 (t, J = 4.9 Hz, 2H), 3.52 (d, J = 6.0 Hz, 2H), 3.40 (t, J = 8.3 Hz, 12H), 3.24 (s, 2H), 1.78–1.85 (m, 12H), 1.43 (td, J = 14.9, 7.4 Hz, 12H), 0.93 (t, J = 7.4 Hz, 18H) ^13^C-NMR (101 MHz, acetone) δ 73.18, 72.59, 70.88, 65.84, 58.57, 54.06, 26.39, 20.29, 13.73 HR-ESI-TOF-MS *m*/*z* 319.4210 (calcd *m*/*z* 319.4274 for B_12_Cl_11_C_5_H_10_O_3_ [M]^2−^).

###### *[Na^+^]_2_[B_12_Cl_11_O(C_2_H_4_O)_2_CH_3_^2−^] (Na)_2_***•9** 

White solid; Yield: 82% mp: 217 °C; B{^1^H} NMR (128.00 MHz, acetone-*d_6_*) δ –7.57 (s, 1B), –13.57 (s, 11B) ^1^H{^11^B} NMR (400.00 MHz, acetone-*d_6_*) δ 4.26 (s, 2H), 3.72 (t, J = 4.6 Hz, 2H), 3.67 (t, J = 4.9 Hz, 2H), 3.60 (t, J = 4.6 Hz, 2H), 3.38 (s, 3H) ^13^C-NMR (101 MHz, acetone) δ 73.22, 72.63, 70.92, 65.88, 58.62 HR-ESI-TOF-MS *m*/*z* 319.4299 (calcd *m*/*z* 319.4274 for B_12_Cl_11_C_5_H_10_O_3_ [M]^2−^).

#### 3.3.5. Solubility of Fluorescein Derivatives

A fluorescein derivative was stirred in water (c.a. 300 μL) at room temperature until saturation was confirmed visually by the presence of undissolved solids. The saturated solution was then filtered to remove any remaining solids. The filtrate was transferred into pre-weighed glass vials, which were immediately weighed to determine the total amount of cesium salt and solvent. The solvent was then evaporated, and the residue was dried under vacuum (1.0 × 10^−1^ hPa, room temperature, 6 h). The residue was weighed to calculate the amount of the sample and the volume of solvent used.

### 3.4. Materials and Methods for Cell Viability Assay

#### 3.4.1. Cell Culture and Imaging

HeLa cells were seeded on 48 well plates (Hounisen, Skanderborg, Denmark, 83.3923) and cultured in DMEM (Thermofisher, Waltham, MA, USA, 10566016) with 15% Fetal Bovine Serum (FBS) and 1000 U/mL of Penicillin–Streptomycin (P/S) (Thermofisher, 15140122) until they became approximately 80% confluent. The boron clusters and fluorophores were diluted in HKR buffer (5 mM of HEPES pH 7.5, 137 mM of NaCl, 2.68 mM of KCl, 2.05 mM of MgCl_2_, 1.8 mM of CaCl_2_, sterilized by filter) and incubated for 15 min at room temperature. After Hela cells were rinsed with HKR buffer, the boron cluster and DAPI mixture were added, and cells were incubated for 1.5 h at 37 °C, 5% CO_2_. We carried out imaging using the Zeiss Axio Observer Z1 Apotome 2 (inverted setup), and images were analyzed by ImageJ 1.54f [22].

#### 3.4.2. Cell Viability Assay

HeLa cells were seeded on 48 well plates and incubated in DMEM with 15% FBS and P/S until they became approximately 60% confluent. The boron clusters were diluted in HKR buffer and added to HeLa cells. After 1.5 h of incubation, HeLa cells were cultured in DMEM with 15% FBS and P/S for 24 h. To dissociate HeLa cells, cells were rinsed with PBS and incubated with 0.05% Trypsin EDTA (Thermofisher, 25300054) at 37 °C for 5 min. Subsequently, trypsin was neutralized by adding an equal volume of DMEM with 15% FBS, and cells were completely dissociated by pipetting. After adding an equal volume of trypan blue (Thermofisher, 15250-061), live and dead cells were counted by a hemocytometer. We confirmed that the total cell number did not show a statistically significant difference among groups and quantified the percentage of live cells over the total cell number.

## 4. Conclusions

In conclusion, we demonstrated that covalent attachment of [B_12_Cl_11_O-]^2−^ to fluorescein-type chromophores via a PEG3 linker enhances membrane permeability and water solubility compared to non-covalent approaches using [B_12_Cl_12_]^2−^ (**2**). The covalent conjugate showed efficient cellular uptake and low cytotoxicity, indicating its potential as a drug delivery carrier. These findings suggest that covalent conjugation with superchaotropic boron clusters could be a promising strategy for improving the cellular uptake of hydrophilic and anionic molecules. Future studies are underway in our laboratory to evaluate the efficacy of this covalent conjugation strategy with diverse cargos, aiming to establish its general utility in enhancing membrane permeability.

## Data Availability

Data are contained within the article or Supplementary Material.

## Supplementary Materials

The following supporting information can be downloaded at: https://www.mdpi.com/article/10.3390/molecules29225416/s1, Figure S1: ^1^H (a), ^11^B{^1^H} (b), ^13^C (c) NMR spectra of (HNbutyl_3_)_2_**•7** in acetone-*d_6_*.; Figure S2: ^11^B{^1^H} NMR spectrum of (Na)_2_**•7** in acetone-*d_6_*.; Figure S3: ^1^H (a), ^11^B{^1^H} (b), ^13^C (c) NMR spectra of (Na)_2_**•4** in acetone-*d_6_*.; Figure S4: ^1^H (a), ^11^B{^1^H} (b), ^13^C (c) NMR spectra of (HNbutyl_3_)_2_**•9** in acetone-*d_6_*.
