# Supplementary material for: Enhancing Membrane Permeability of Fluorescein-Type Chromophore Through Covalent Attachment of Chlorinated Dodecaborate
Source: Molecules. 2024 Nov 17;29(22):5416. doi: 10.3390/molecules29225416 (PMC11597110; doi:10.3390/molecules29225416)
Supplement: Supplementary file 1 [file molecules-29-05416-s001.zip › molecules-3293936-supplementary.pdf]

*Supporting Information for*

# Enhancing Membrane Permeability of Fluorescein-type Chromophore through Covalent Attachment of Chlorinated Dodecaborate

**Hibiki Nakamura [1], Satoshi Yamamoto [1], Yumiko K. Kawamura [2], Taro Kitazawa [2,*], Mutsumi Kimura [1,3,*] and Yu Kitazawa [3,*]**

[1] Department of Chemistry and Materials, Faculty of Textile Science and Technology, Shinshu University, Ueda, 386-8567, Japan.

[2] Danish Research Institute of Translational Neuroscience - DANDRITE, Nordic-EMBL Partnership for Molecular Medicine.

[3] Aarhus University, Department of Molecular Biology and Genetics, Universitetsbyen 81, 8000 Aarhus, Denmark.

[4] *Research Initiative for Supra-Materials (RISM), Shinshu University, Ueda, 386-8567, Japan*

## Table of Contents

NMR Spectra of Compounds S-2

# 1. NMR Spectra of Compounds

(a)

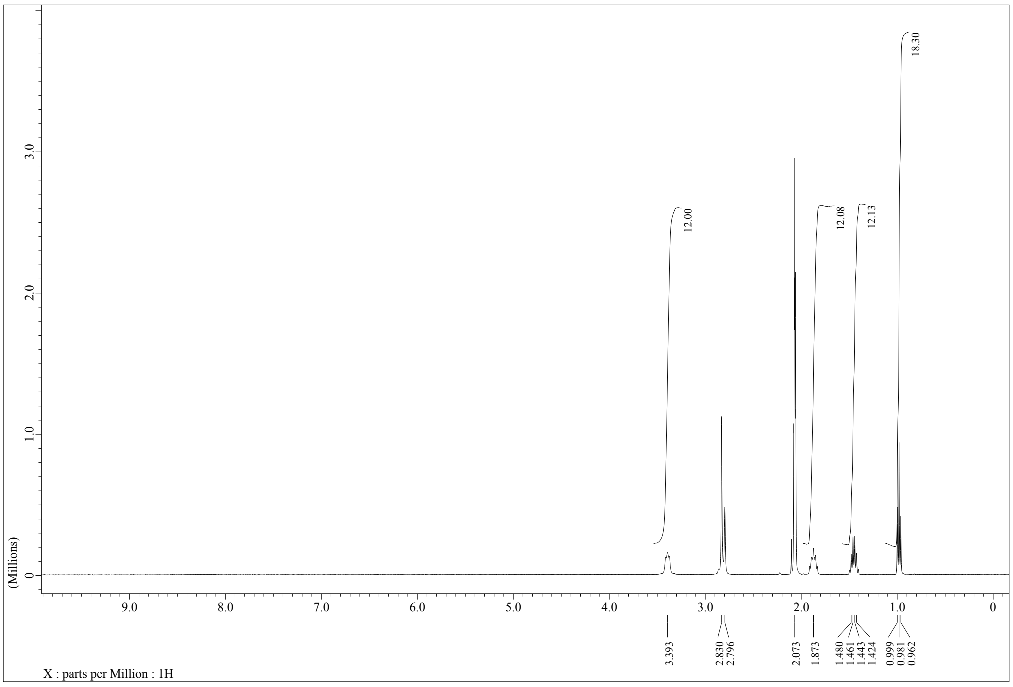


(b)

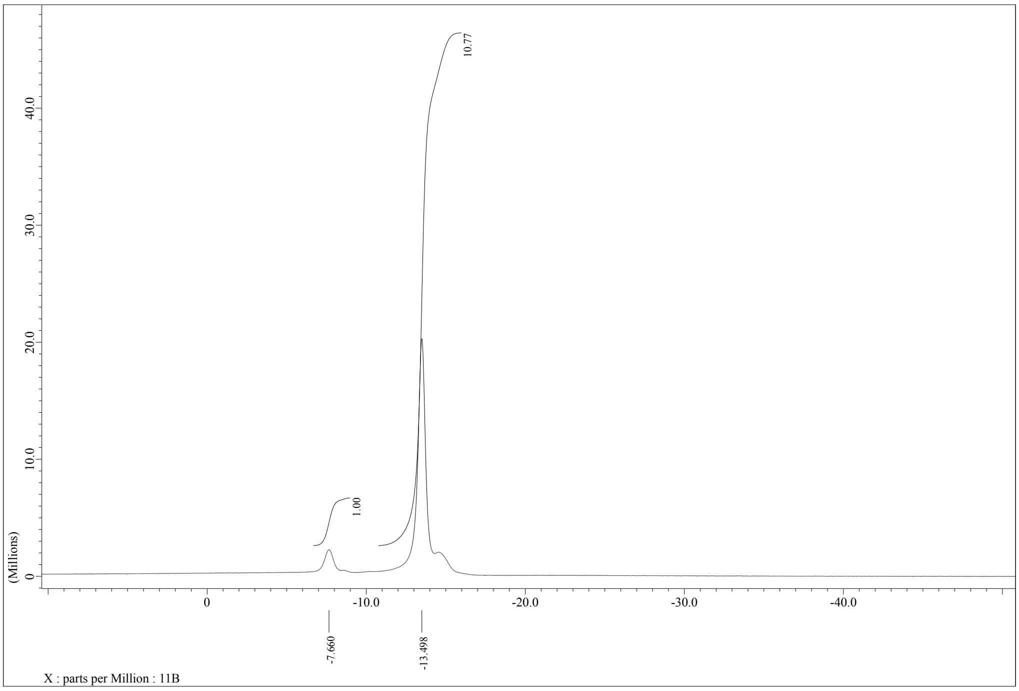

(c)

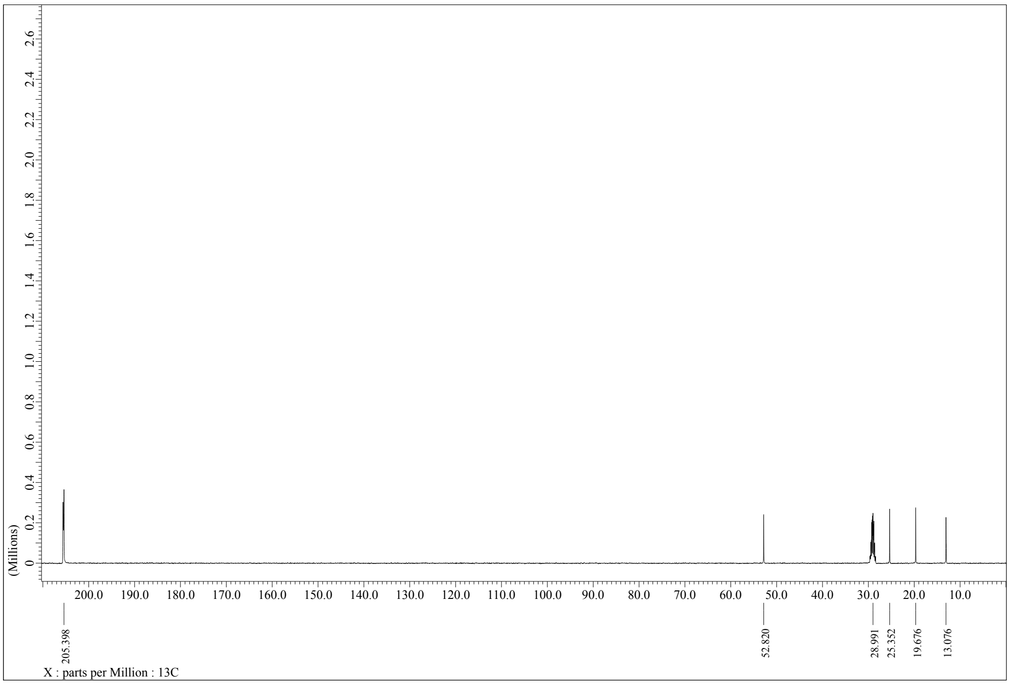


**Figure S1.** $^{1}H$ (a), $^{11}B\{^{1}H\}$ (b), $^{13}C$ (c) NMR spectra of $(HNbutyl_3)_2$•**7** in acetone-$d_6$.

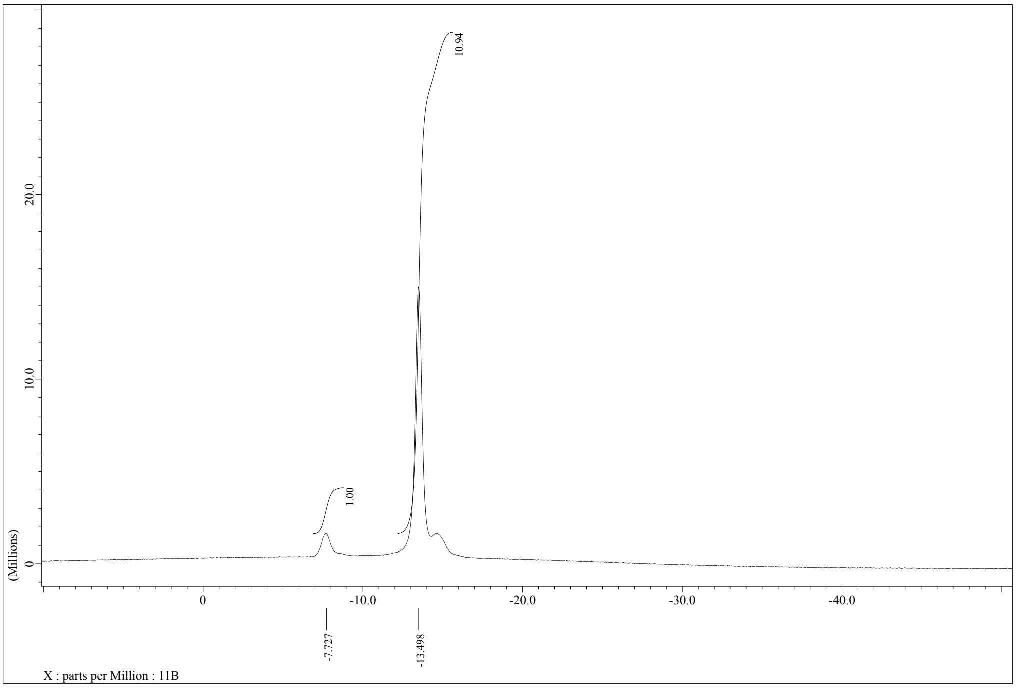


**Figure S2.** $^{11}B\{^{1}H\}$ NMR spectrum of $(Na)_2$•**7** in acetone-$d_6$.

(a)

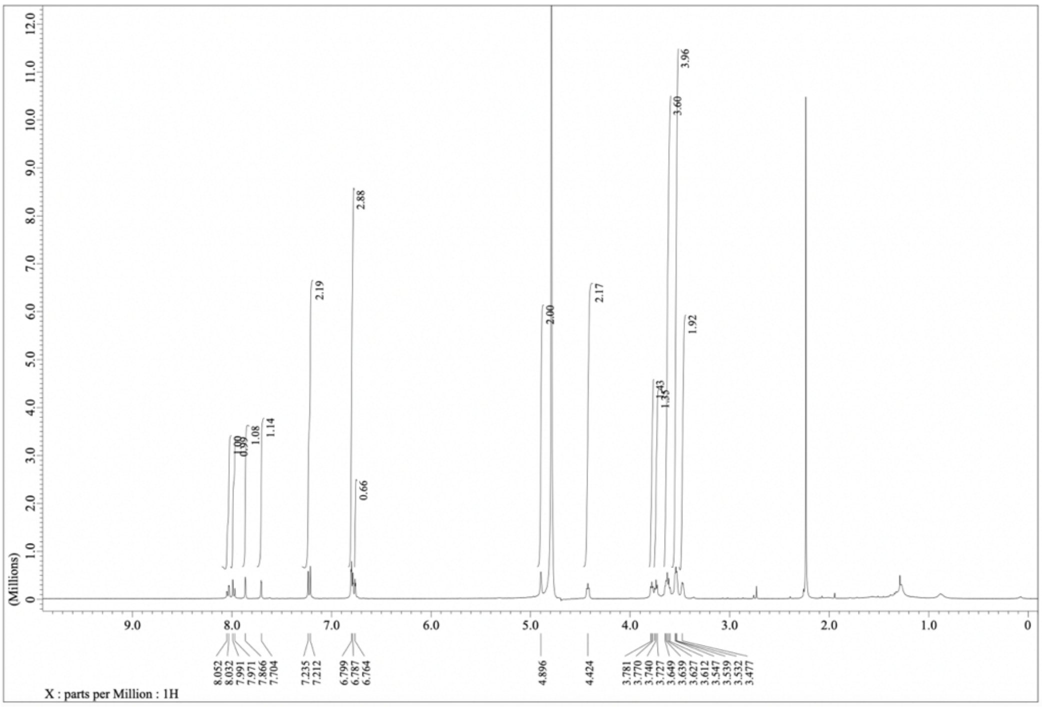
X : parts per Million : 1H
(Millions)

(b)

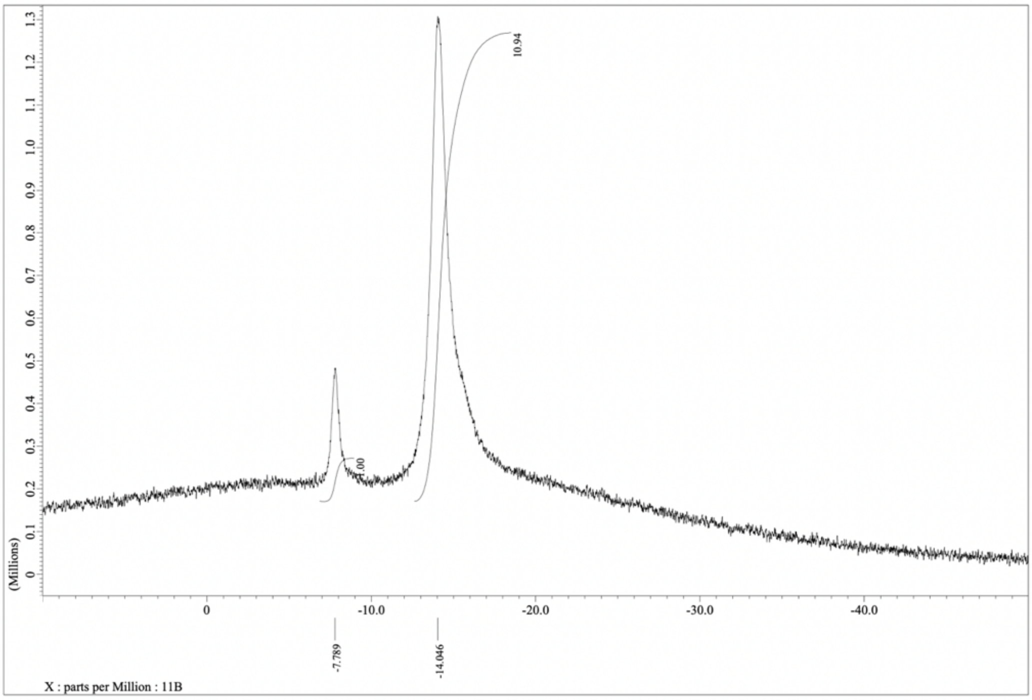
X : parts per Million : 11B
(Millions)
-7.789
-14.046
1.00
10.94

(c)

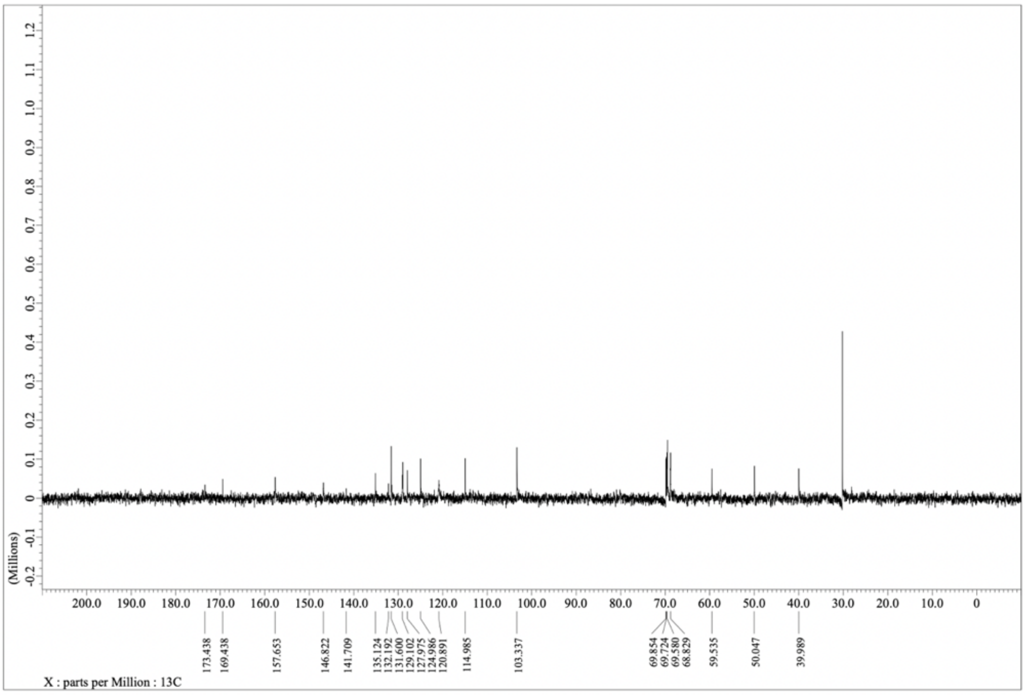

**Figure S3.** $^{1}H$ (a), $^{11}B\{^{1}H\}$ (b), $^{13}C$ (c) NMR spectra of $(Na)_2$•**4** in acetone-$d_6$.

(a)

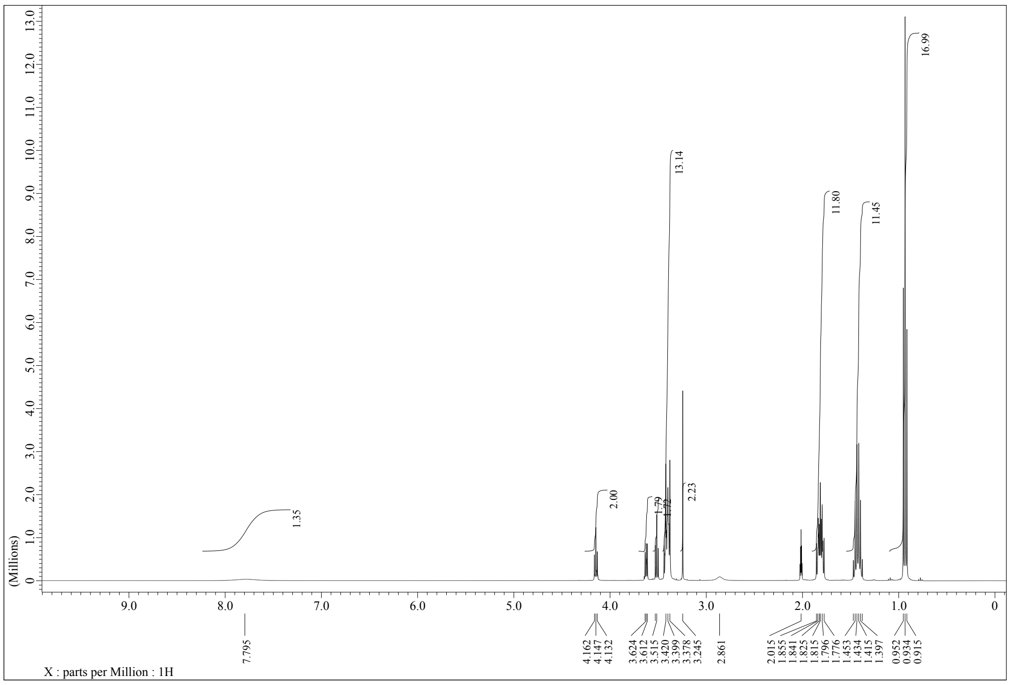

(b)

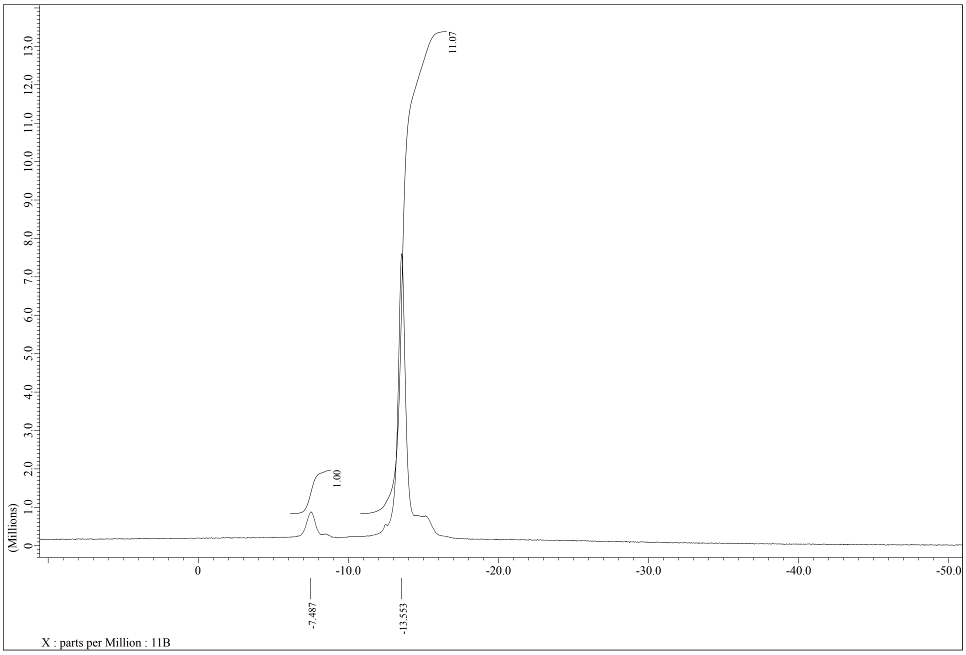


(c)

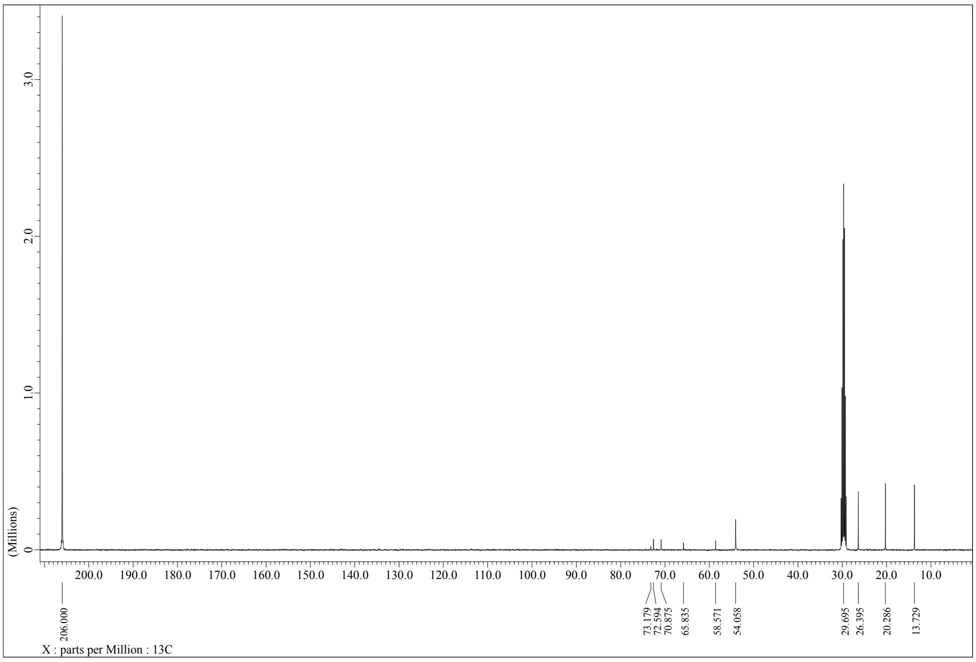


**Figure S4.** $^{1}$H (a), $^{11}$B{$^{1}$H} (b), $^{13}$C (c) NMR spectra of (HNbutyl$_3$)$_2$•**9** in acetone-*d*$_6$.
